# A Deportment Coronet

**Published:** 1914-05-23

**Authors:** 


### A Deportment Coronet.

The glories of the art of deportment have seemed rather neglected since the demise of Mr. Mantalini, Mr. Turveydrop, and Mr. Chadband, but the scientific view of deportment is still engaging attention. In fact, we have received from the inventor, Mr. James S. Collis, of 100 Balham Park Road, S.W., an ingenious contrivance aptly described as a "Deportment Coronet." The apparatus consists of a light cane horseshoe, neatly padded, which is placed horizontally on the forehead and temples, and a small wire contrivance held vertically in two holes in the front of the coronet. This portion of the apparatus contains a freely movable crossbar, which remains in a certain position so long as the head of the wearer is held erect, but slides forwards and downwards, making a perceptible click as it falls, when the head is allowed to decline. The total weight of the coronet does not exceed 1¼ oz., and so headache is not likely to be caused. Worn for a quarter of an hour several times a day it should, as the inventor claims, assist in imparting grace of carriage and prevent that slouching and drooping of the shoulders which is not only displeasing to the eye but inimical to good health and physique, destroying as it does the dignity, if not the value, of the erect posture. Parents and teachers might well consider the advantages of this simple apparatus, which is obtainable from the inventor at the price of 2s. 6d.

---

Falkirk.—The Dean of Guild Court has granted permission to the Falkirk Infirmary management to extend the south ward of the infirmary. It is proposed to erect a verandah and a sanitary wing capable of holding sixteen beds. The cost is estimated at £1,000. Mr. Black is the architect in charge.

